# Quantitative gait analysis in mild cognitive impairment, dementia, and cognitively intact individuals: a cross-sectional case–control study

**DOI:** 10.1186/s12877-022-03405-9

**Published:** 2022-09-23

**Authors:** Sunee Bovonsunthonchai, Roongtiwa Vachalathiti, Vimonwan Hiengkaew, Mon S. Bryant, Jim Richards, Vorapun Senanarong

**Affiliations:** 1 Faculty of Physical Therapy, Mahidol University, Nakhon Pathom, Thailand; 2 Department of Physical Medicine and Rehabilitation, Baylor College of Medicine, Houston, TX USA; 3 Allied Health Research Unit, University of Central Lancashire, Preston, UK; 4 Division of Neurology, Faculty of Medicine Siriraj Hospital, Mahidol University, Bangkok, Thailand

**Keywords:** Gait, Mild cognitive impairment, Dementia, Classification

## Abstract

**Background:**

Cognitive age-related decline is linked to dementia development and gait has been proposed to measure the change in brain function. This study aimed to investigate if spatiotemporal gait variables could be used to differentiate between the three cognitive status groups.

**Methods:**

Ninety-three older adults were screened and classified into three groups; mild cognitive impairment (MCI) (*n* = 32), dementia (*n* = 31), and a cognitively intact (*n* = 30). Spatiotemporal gait variables were assessed under single- and dual-tasks using an objective platform system. Effects of cognitive status and walking task were analyzed using a two-way ANCOVA. Sub-comparisons for between- and within-group were performed by one-way ANCOVA and Paired t-tests. Area Under the Curve (AUC) of Receiver Operating Characteristics (ROC) was used to discriminate between three groups on gait variables.

**Results:**

There were significant effects (*P* < 0.05) of cognitive status during both single and dual-task walking in several variables between the MCI and dementia and between dementia and cognitively intact groups, while no difference was seen between the MCI and cognitively intact groups. A large differentiation effect between the groups was found for step length, stride length, and gait speed during both conditions of walking.

**Conclusions:**

Spatiotemporal gait variables showed discriminative ability between dementia and cognitively intact groups in both single and dual-tasks. This suggests that gait could potentially be used as a clinical differentiation marker for individuals with cognitive problems.

## Background

Dementia affects approximately 47 million people worldwide, with 10 million new cases reported each year [1]. In East Asia, the prevalence of dementia is increasing which has been shown to be associated with cardiovascular risk factors [2] or exposure to air pollution [3]. In contrast, the declination of dementia incidence was reported in high-income countries such as the United States, the United Kingdom, Sweden, the Netherlands, France, and Iceland [2, 4]. In Thailand, the majority of cases were caused by Alzheimer's disease (AD), followed by vascular dementia, dementia with Lewy bodies, frontotemporal dementia, and others [5]. Dementia is a group of different progressive diseases that currently has no cure and is arguably the most pressing public health challenge [2, 6]. It is a syndrome that primarily affects older people and is characterized by a decline in cognitive performance that affects the individual's ability related to cognitive and physical functions [1, 7–10]. Dementia and falls were reported as the top five causes of disability-adjusted life-years (DALYs) in people aged 75 and older [11]. This is an important cause of disability and dependency and has an impact on family and carers' physical, psychological and social well-being [1, 6, 12]. Among the various problems faced, individuals with dementia frequently experience gait and balance issues, which contribute to a significantly increased risk of falling [13–16], leading to fractures, hospitalization, functional dependence, or death [1, 14, 15].

Early detection of dementia is frequently challenging, as the functional deficits required for such diagnoses can be caused or exacerbated by comorbidities and social circumstances [17–19]. Progression of the disease escalates over time if individuals are misdiagnosed, which leads to inappropriate clinical management [10, 20–22]. As a result, it is crucial to detect dementia quickly and accurately, as well as to monitor the disease progression. substantial evidence demonstrates that timely diagnosis and treatment can prevent the progression of dementia or its consequences [16, 21, 23, 24]. A non-invasive, sensitive, and cost-effective marker is therefore required to help identify and classify individuals with cognitive decline [25].

Gait performance has been studied to determine its efficacy in screening individuals with cognitive dysfunction. Clinical and epidemiologic evidence supports the concept that gait is interrelated with cognitive ability in the elderly [26–29]. It has been reported that healthy younger individuals can preserve their primary walking when faced with a secondary cognitive task, whereas older adults have difficulty in preserving their gait performance during dual tasks, and this capability is greatly reduced in those with cognitive dysfunction [21, 30–32]. An attempt to investigate the relationship between gait and cognition not only provides a better understanding of function but also provides an assessment of safety during movement. Decreased ability to walk during a single- or cognitive dual-task has been reported to be a contributing factor to falls in older adults and individuals with cognitive decline [33, 34]. Dual-task gait assessment, where individuals have to perform a secondary attentional challenging task while walking, was shown to be more challenging as the tasks interfere with each other and divide the cortical control resources of the brain [35]. Gait modifications, such as reduced gait speed, rhythm, sway, instability, or stopping walking, can occur while performing the second task together, especially in individuals with cognitive decline [35–37]. In addition, a meta-analysis has demonstrated the importance of gait and cognition factors with the dual decline of gait speed and memory having the highest predictive risk of developing dementia among older individuals [31].

The role of using gait in classifying individuals with or without cognitive decline has been shown in the studies [16, 38–47]. However, based on the different reports previously, the results varied from study to study. This may be a result of differences in participants' characteristics, measuring equipment, testing variables, and the walking data collection process. Yet most of the reported findings came from western countries [40–47] and less was done in Asian countries and reported on a small sample size [16, 38, 39]. Different biological structures, cultures, living behaviors, quality of life, pollution and environment, and education all could influence gait and cognitive profiles. Hence, data from the studies on different populations, especially people in Asian countries, are still needed. In addition, which gait variables are the most sensitive and practical to discriminate between groups of individuals with different cognitive levels is needed. This study therefore explored the use of gait profiles to differentiate people with different cognitive profiles within a South East Asian population.

Therefore, this study aimed to conduct a case–control study to differentiate individuals with and without cognitive impairment. We hypothesized that gait variables would be sensitive enough to differentiate between the groups with and without cognitive impairment, and these would be easier to detect under dual-task conditions.

## Methods

### Study design and recruitment

A cross-sectional case–control study with a convenience sampling method was used. Individuals living with MCI and dementia from the Memory Clinic, Siriraj hospital, Bangkok, Thailand were invited to participate in the study. The individuals who were cognitively intact were recruited from able-bodied individuals who live in Bangkok and metropolitan areas through word-of-mouth. All participants voluntarily participated in the study and were informed about the research objectives, benefits, and details before data collection. Once they understood and agreed to participate, project staff made an appointment for the date and time to measure gait and other related data.

### Participants

Participants in the MCI, dementia, and cognitively intact groups were subjectively and physically examined by neurologists. Participants were community-dwelling middle and older adults with or without a diagnosis of MCI or dementia. Diagnosis of MCI and dementia was performed through the consensus of expert neurologists and psychologists who have routinely worked at Siriraj hospital based on the Diagnostic and Statistical Manual of Mental Disorders, 5^th^ edition (DSM-5) criteria [48] and the National Institute of Aging-Alzheimer’s Associaton criteria [49]. The DSM-5 criteria indicate the key symptoms used to distinguish MCI from dementia as the evidence of modest cognitive decline from a previous level of performance in one or more of the listed domains (cognitive deficits are not interfering with the independence of instrumental activities of daily living, cognitive deficits do not occur exclusively in the context of a delirium, and cognitive deficits are not primarily attributable to another mental disorder). The latter criteria indicate the preservation of independence in functional abilities and lack of significant impairment in social or occupational functioning for the MCI. Sub-types of MCI were defined based on the presence or absence of memory difficulties (amnestic and non-amnestic MCI). For the cognitively intact individuals, they had normal cognitive performance and were independent in their daily living activities. The exclusion criteria for all groups of participants were; severe cognitive function, unable to follow commands and instructions, serious infection, psychiatric disorder, epilepsy, and any other conditions that may affect the performance of the walking tasks such as severe pain, being blind, or deaf.

### Procedure

The study was conducted at the Neuro Computational Intelligence for Neuro-Cognitive disorders Laboratory (NN lab), Siriraj Medical Research Center (SiMR), Faculty of Medicine, Siriraj Hospital, Mahidol University, Bangkok, Thailand. Demographic data recorded included; sex, age, body weight and height, body mass index (BMI), year of education, cognitive function using the Montreal Cognitive Assessment (MoCA), disease duration of the cognitive decline, types, and underlying diseases. MoCA is one of the cognitive tools which was developed by Nasreddine et al. [50]. The total possible score of MoCA ranges from 0–30 scores assessing several cognitive domains (visuospatial/executive, naming, memory, attention, language, abstraction, delayed recall, and orientation). It has been tested for sensitivity and is widely used to investigate cognitive status [50–53]. In addition, the Functional Ambulatory Category (FAC) is a six-point scale used to evaluate functional gait status. The scale assesses the level of support that the patient requires during walking, regardless of whether or not an assistive device is used [54]. The ambulatory status could be classified into six levels, ranging from non-functional ambulator (FAC = 0) to independent ambulator (FAC = 5). This is a quick visual measurement of functional gait ability and is administered in individuals with dementia [55].

Spatiotemporal gait variables were then collected at 100 Hz using the Force Distribution Measurement (FDM) platform which had a length of 307 cm and width of 60.5 cm (Zebris Medical GmbH, Germany). The platform was installed in the middle part of a 5 m walkway and was synchronized with a video camera (SYNCCam) placed in front of the participants to check walking patterns for each trial. The FDM platform is widely used for gait assessment in various neurological conditions and was used as a gold standard to another proposed tool [56–59]. To remove the effect of the acceleration and deceleration phases of walking, gait data were recorded from the middle part of the walkway. Spatiotemporal gait data were extracted and analyzed using the WinFDM software (Zebris Medical GmbH, Germany, version 1.18.48).

For the single-task condition, the participants were asked to walk at their own usual walking pace on the walking platform. For the dual-task condition, to monitor the compliance of the testing, participants were asked to count a number backward by one out loud while walking at their usual walking pace [39, 40]. A physical therapist walked slightly behind the participants to prevent slips, trips, and falls during the data collection. Three successful trials were recorded under each condition and the average of the three trials was used in the data analysis.

### Outcomes

Spatiotemporal gait variables were used for the comparison and discrimination analyses. These included; foot rotation angle (deg), step length (cm), stride length (cm), step width (cm), stance phase as a percentage of the gait cycle (%GC)), loading response (%GC), single support phase (%GC), pre-swing (%GC), swing phase (%GC), double support phase (%GC), step time (s), stride time (s), cadence (steps/min), and gait speed (m/s).

### Data analysis

SPSS version 26.0 (IBM Corp, USA) was used for all analyses. Participant characteristics and clinical variables were descriptively analyzed. A two-way ANCOVA was used to determine the main effects and interaction effects of the cognitive status groups (MCI, dementia, and cognitively intact) and walking task (single and dual). Comparisons of gait variables between the MCI, dementia, and cognitively intact groups during single- and dual-task walking were explored using one-way ANCOVA tests. Regarding age, sex, and BMI are the biological important factors that could affect gait variables, so these factors were used as the covariates in the analysis and the adjusted mean values were reported.

Comparisons of gait variables between single- and dual-task walking were performed within the groups using the Paired t-test. A Bonferroni correction for multiple comparisons was used for the two-way ANCOVA and one-way ANCOVA tests, and adjusted *p*-values were used for the Paired t-test analysis by dividing the *p*-value by the number of variables being tested or number of the test (*P* < 0.05/14 or *P* < 0.004). The area under the curve (AUC) of the Receiver Operating Characteristics (ROC) was also used to determine the accuracy of discrete gait parameters between the MCI, dementia, and cognitively intact groups. Accuracy of AUC 0.5 was considered as no discrimination, 0.7–0.8 was considered acceptable, 0.8–0.9 was considered excellent, and more than 0.9 was considered outstanding [60].

## Results

### Participant characteristics

Participant characteristics are presented in Table 1. Ninety-three participants (70 females and 23 males) were included in this study and were classified into MCI (*n* = 32), dementia (*n* = 31), and cognitively intact (*n* = 30) groups. No differences were found between the groups for sex, body weight, height, and body mass index, with an age range of 63.6 − 71.8 years and mean body weight and height of 56.7 − 63.0 kg and 155.9 − 158.4 cm, respectively. A greater number of females were seen in all three groups (68.75 − 86.67%). Age, year of education, and MoCA showed significant differences between the groups (*P* < 0.05), with the cognitively intact group being the youngest (63.6 years) and the dementia group being the oldest (71.8 years). The cognitively intact group had the highest year of education (15.9 years) and the dementia group had the lowest (10.4 years). The greatest median MoCA score was seen in the cognitively intact group (28 scores), followed by the MCI group (25 scores), and the lowest was seen in the dementia group (14 scores). The mean onset duration of MCI was 3.38 years, with a similar number of individuals with amnestic and non-amnestic types (*n* = 15 and *n* = 17), respectively. The mean onset duration of dementia was 4.68 years, with the most common type being Alzheimer's disease (AD) (*n* = 20), followed by Frontotemporal Lobar Degeneration (FTLD) (*n* = 6), Dementia with Lewy Bodies (DLB) (*n* = 3), and Vascular dementia (VaD) (*n* = 2). Hypertension was the only underlying disease that showed a difference between groups, with the dementia group having the highest number of individuals (*n* = 19 or 65.52%), followed by the MCI group (*n* = 17 or 60.71%), with only 15.3% in the cognitively intact group (*n* = 4). No other differences were found for underlying diseases between the groups (*P* > 0.05). All participants in this study could walk on the surface, as shown with the FAC score of 4 and 5 scores. It was found that most individuals with MCI and dementia were able to walk independently (FAC = 5).

**Table 1:** Participant characteristics

Variables	MCI (*n* = 32)	Dementia (*n* = 31)	Cognitively intact (*n* = 30)	Total (*n* = 93)	*P*-value
Sex: Female/Male ^a^, n (%)	22 (68.75)/10 (31.25)	22 (70.97)/9 (29.03)	26 (86.67)/4 (13.33)	70 (75.27)/23 (24.73)	0.212
Age^b^ (years), mean ± SD	69.91 ± 6.96	71.81 ± 9.46	63.57 ± 4.78	68.49 ± 8.06	** < 0.001**
Weight ^b^ (kg), mean ± SD	63.03 ± 11.39	57.68 ± 11.48	56.73 ± 11.63	59.22 ± 11.71	0.070
Height^b^ (cm), mean ± SD	158.41 ± 7.54	158.06 ± 9.59	155.90 ± 6.73	157.48 ± 8.04	0.422
Body mass index ^b^ (kg/m^2^)	25.02 ± 3.52	23.01 ± 3.79	23.30 ± 4.29	23.80 ± 3.93	0.089
Years of education ^b^ (years), mean ± SD	13.16 ± 5.19	10.35 ± 6.25	15.87 ± 3.07	13.10 ± 5.46	** < 0.001**
MoCA ^a^ (scores), Median (IQR)	25 (21 − 27)	14 (10 − 20)	28 (26 − 29)	25 (18 − 28)	** < 0.001**
Disease duration ^c^ (years), mean ± SD	3.38 ± 3.83	4.68 ± 3.25	N/A	4.02 ± 3.59	0.151
Type, n (%) amnestic MCI/non-amnestic MCI or AD/FTLD/DLB/VaD	15 (46.88)/17(53.1)	20 (64.52)/6 (19.35)/ 3 (9.68)/2 (6.45)	N/A	N/A	N/A
Comorbidity ^a^: yes/no, n (%) ^**^					
Hypertension	19 (65.52)/10 (34.48)	17 (60.71)/11 (39.29)	4 (15.28)/22 (84.62)	40 (48.19)/43 (51.81)	** < 0.001**
Diabetes mellitus	9 (31.03)/20 (68.97)	7 (25.00)/21 (75.00)	3 (11.54)/23 (88.46)	19 (22.89)/64 (77.11)	0.221
Heart disease	3 (10.34)/26 (89.66)	2 (7.14)/26 (92.86)	1 (3.85)/25 (96.15)	6 (7.23)/77 (92.77)	0.653
Cerebrovascular disease	6 (20.69)/23 (79.31)	3 (10.71)/25 (89.29)	1 (3.85)/25 (96.15)	10 (12.05)/73 (87.95)	0.158
Thyroid disease	4 (13.79)/25 (86.21)	3 (10.71)/25 (89.29)	3 (11.54)/23 (88.46)	10 (12.05)/73 (87.95)	0.935
FAC ^a^ (scores), n (%) 4/5	2 (6.25)/ 30 (93.75)	7 (22.58)/24 (77.42)	0 (0)/100 (100)	9 (9.68)/84 (90.32)	0.009

*MCI* Mild cognitive impairment, *MoCA* Montreal Cognitive Assessment, *AD* Alzheimer's disease, *FTLD* Frontotemporal Lobar Degeneration, *DLB* Dementia with Lewy Bodies, *VaD* Vascular dementia, *FAC* Functional Ambulatory Category, *N/A* not assessment; Statistical significance tested by the

^a^Kruskal Wallis

^b^One way ANOVA, and

^c^Independent sample t-test at *P* < 0.05

^*^ Missing data (4 for dementia)

^**^ Missing data (3 for the MCI and dementia each and 4 for cognitively intact)

### The main effects and interactions of cognitive status and walking task on gait variables

The two-way ANCOVA demonstrated significant interaction effects for cognitive status (MCI, dementia, and cognitively intact) and walking task (single and dual) including step time (*P* = 0.037, η^2^_*p*_ = 0.038) and stride time (*P* = 0.036, η^2^_*p*_ = 0.038). Significant main effects of cognitive status were found in several variables; step length (*p* < 0.001, η^2^_*p*_ = 0.171), stride length (*p* < 0.001, η^2^_*p*_ = 0.171), step width (*P* = 0.012, η^2^_*p*_ = 0.050), stance phase (*P* < 0.001, η^2^_*p*_ = 0.114), loading response (*P* < 0.001, η^2^_*p*_ = 0.117), single support phase (*P* < 0.001, η^2^_*p*_ = 0.098), pre-swing (*P* < 0.001, η^2^_*p*_ = 0.116), swing phase (*P* < 0.001, η^2^_*p*_ = 0.114), double support phase (*P* < 0.001, η^2^_*p*_ = 0.112), step time (*P* = 0.005, η^2^_*p*_ = 0.060), stride time (*P* = 0.005, η^2^_*p*_ = 0.061), cadence (*P* = 0.002, η^2^_*p*_ = 0.069), and gait speed (*P* < 0.001, η^2^_*p*_ = 0.152).

Significant main effects of walking tasks were found in almost all gait variables including; step length (*P* = 0.001, η^2^_*p*_ = 0.061), stride length (*P* = 0.001, η^2^_*p*_ = 0.061), stance phase (*P* = 0.001, η^2^_*p*_ = 0.062), loading response (*P* < 0.001, η^2^_*p*_ = 0.072), single support phase (*P* < 0.001, η^2^_*p*_ = 0.079), pre-swing (*P* < 0.001, η^2^_*p*_ = 0.076), swing phase (*P* = 0.001, η^2^_*p*_ = 0.062), double support phase (*P* < 0.001, η^2^_*p*_ = 0.072), step time (*P* < 0.001, η^2^_*p*_ = 0.258), stride time (*P* < 0.001, η^2^_*p*_ = 0.259), cadence, (*P* < 0.001, η^2^_*p*_ = 0.284), and gait speed (*P* < 0.001, η^2^_*p*_ = 0.185).

### Comparison of gait variables between the groups under the single and dual walking tasks

As shown in Table 2, during single-task walking, there were significant differences (*P* < 0.05) between the groups for step length, stride length, stance phase, loading response, single support phase, pre-swing, swing phase, double support phase, and gait speed. The post-hoc test showed differences between the MCI and dementia groups as well as between dementia and cognitively intact groups. During dual-task walking, significant differences were seen between the groups in all gait variables (*P* < 0.05), except for foot rotation angle. The post-hoc test showed differences between the MCI and dementia groups as well as between dementia and cognitively intact groups in almost variables.

**Table 2:** Gait variables of the MCI (*n* = 32), dementia (*n* = 31), and cognitively intact (*n* = 30) groups in single- and dual-task walking (Adjusted means ± standard error)

Gait variables	Single-task walking						Dual-task walking						*P*-value ^**^		
	**MCI**	**Dementia**	**Cognitively intact**	**η**^**2**^_***p***_	*P*-value ^*^	**Post-hoc**	**MCI**	**Dementia†**	**Cognitively intact**	**η**^**2**^_***p***_	*P*-value ^*^	**Post-hoc**	**MCI**	**Dementia**	**Cognitively intact**
Foot rotation angle (deg)	9.18 ± 1.04	11.36 ± 1.08	9.10 ± 1.14	0.031	0.258	N/A	9.38 ± 1.13	12.07 ± 1.27	9.62 ± 1.23	0.035	0.237	N/A	0.441	0.026	0.233
Step length (cm)	48.52 ± 1.36	42.58 ± 1.41	51.94 ± 1.49	0.189	** < 0.001**	a, c	44.58 ± 1.62	38.30 ± 1.82	48.38 ± 1.77	0.155	**0.001**	a, c	** < 0.001**	** < 0.001**	** < 0.001**
Stride length (cm)	97.04 ± 2.72	85.15 ± 2.81	103.88 ± 2.97	0.189	** < 0.001**	a, c	89.17 ± 3.24	76.61 ± 3.64	96.76 ± 3.54	0.155	**0.001**	a, c	** < 0.001**	** < 0.001**	** < 0.001**
Step width (cm)	11.59 ± 0.47	12.04 ± 0.49	10.65 ± 0.52	0.039	0.174	N/A	12.09 ± 0.56	13.14 ± 0.62	11.07 ± 0.61	0.060	0.078	N/A	0.038	** < 0.001**	0.827
Stance phase (%GC)	67.24 ± 0.57	69.78 ± 0.59	67.05 ± 0.63	0.126	**0.003**	a, c	69.08 ± 0.80	72.23 ± 0.89	68.51 ± 0.87	0.110	**0.008**	a, c	**0.002**	** < 0.001**	0.043
Loading response (%GC)	17.11 ± 0.58	19.78 ± 0.60	17.09 ± 0.63	0.130	**0.002**	a, c	19.12 ± 0.79	22.35 ± 0.88	18.68 ± 0.86	0.113	**0.007**	a, c	**0.001**	** < 0.001**	0.016
Single support phase (%GC)	32.89 ± 0.57	30.28 ± 0.59	32.94 ± 0.62	0.131	**0.002**	a, c	30.82 ± 0.77	27.68 ± 0.87	31.23 ± 0.85	0.110	**0.008**	a, c	**0.001**	** < 0.001**	0.007
Pre-swing (%GC)	17.00 ± 0.56	19.55 ± 0.58	16.87 ± 0.61	0.131	**0.002**	a, c	19.07 ± 0.79	22.05 ± 0.88	18.55 ± 0.86	0.101	**0.013**	a, c	**0.001**	** < 0.001**	0.005
Swing phase (%GC)	32.76 ± 0.57	30.22 ± 0.59	32.95 ± 0.63	0.126	**0.003**	a, c	30.92 ± 0.80	27.77 ± 0.89	31.49 ± 0.87	0.110	**0.008**	a, c	**0.002**	** < 0.001**	0.042
Double support phase (%GC)	34.03 ± 1.14	39.27 ± 1.18	33.79 ± 1.24	0.133	**0.002**	a, c	38.06 ± 1.58	44.17 ± 1.77	37.17 ± 1.72	0.103	**0.012**	a, c	**0.001**	** < 0.001**	0.007
Step time (s)	0.59 ± 0.02	0.63 ± 0.02	0.58 ± 0.02	0.053	0.092	N/A	0.76 ± 0.04	0.88 ± 0.04	0.73 ± 0.04	0.075	**0.042**	N/D	** < 0.001**	** < 0.001**	** < 0.001**
Stride time (s)	1.17 ± 0.03	1.26 ± 0.03	1.16 ± 0.03	0.053	0.093	N/A	1.51 ± 0.08	1.75 ± 0.09	1.46 ± 0.08	0.076	**0.039**	N/D	** < 0.001**	** < 0.001**	** < 0.001**
Cadence (steps/min)	103.81 ± 2.34	97.70 ± 2.42	104.55 ± 2.56	0.050	0.108	N/A	85.80 ± 3.06	75.66 ± 3.43	88.33 ± 3.34	0.085	**0.026**	c	** < 0.001**	** < 0.001**	** < 0.001**
Gait Speed (m/s)	0.84 ± 0.04	0.69 ± 0.04	0.89 ± 0.04	0.147	**0.001**	a, c	0.65 ± 0.04	0.50 ± 0.04	0.73 ± 0.04	0.156	**0.001**	a, c	** < 0.001**	** < 0.001**	** < 0.001**

%GC: Percent of gait cycle; η^2^_*p*_: Partial Eta square; **†**number of participants was reduced to *n* = 26 due to some individuals being unable to perform the dual-task

^*^ P-value tested by the one way ANCOVA using age, sex, and BMI as covariates, followed by the Bonferroni post hoc correction between a: MCI and Dementia, b: MCI and Cognitively intact, and c: Dementia and Cognitively intact; N/A: not assessed; N/D: no difference

^**^ P-value tested by the Paired t-test between single- and dual-task walking in each group of the participants; Bold values demonstrated significant difference at *P* < 0.05 for One-way ANCOVA and *P* < 0.004 for Paired t-test

### Comparisons of gait variables between single and dual-task walking within each group

Paired t-tests were used to compare gait variables between single and dual-task walking within each group separately as shown in Table 2. A Bonferroni correction was used to adjust the significance level to allow for the number of comparisons. So, the *P*-value was set at < 0.05/14 or *P* < 0.004. Significant differences were found in almost gait variables (*P* < 0.004) between single- and dual-task walking in the MCI and dementia groups, with exception of the foot rotation angle. For the cognitively intact group, differences were seen only in step length, stride length, step time, stride time, cadence, and gait speed.

### Discriminating gait characteristics between the MCI, dementia, and cognitively intact groups

Discriminating gait characteristics between the groups are presented in Table 3. Based on the accuracy value criteria of the AUC of ≥ 0.7 with a statistical significance of < 0.05, it was found that a single spatiotemporal gait variable differentiated between dementia and cognitively intact groups only. For the single-task walking, three variables including step length, stride length, and gait speed provided excellent discrimination levels between the groups (AUC = 0.823–0.849). During dual-task walking, excellent discrimination was found in the same set of gait variables similar to single-task walking and single support phase (AUC = 0.813–0.823), with the highest discrimination ability shown by gait speed.

**Table 3:** Area under the curve (AUC) and 95% confidence interval (CI) of gait variables between the groups during single- and dual-task walking

Gait variables	MCI vs Dementia				MCI vs Cognitively intact				Dementia vs Cognitively intact			
	**AUC**	**95% CI**		***p*-value**	**AUC**	**95% CI**		***p*-value**	**AUC**	**95% CI**		***p*-value**
		**Lower bound**	**Upper bound**			**Lower bound**	**Upper bound**			**Lower bound**	**Upper bound**	
**Single-task walking:**												
Foot rotation angle (deg)	0.586	0.444	0.727	0.243	0.574	0.429	0.719	0.317	0.343	0.204	0.482	0.035
Step length (cm)	0.309	0.177	0.441	0.009	0.287	0.159	0.416	0.004	**0.849**	**0.752**	**0.946**	** < 0.001**
Stride length (cm)	0.309	0.177	0.441	0.009	0.287	0.159	0.416	0.004	**0.848**	**0.750**	**0.945**	** < 0.001**
Step width (cm)	0.561	0.418	0.705	0.402	0.676	0.541	0.811	0.017	0.289	0.158	0.419	0.005
Stance phase (%GC)	0.671	0.537	0.804	0.020	0.603	0.462	0.744	0.163	0.228	0.113	0.343	< 0.001
Loading response (%GC)	0.679	0.548	0.811	0.014	0.581	0.438	0.724	0.272	0.230	0.114	0.346	< 0.001
Single support phase (%GC)	0.307	0.178	0.437	0.009	0.408	0.266	0.550	0.215	**0.778**	**0.664**	**0.892**	** < 0.001**
Pre-Swing (%GC)	0.686	0.555	0.818	0.011	0.595	0.453	0.737	0.200	0.220	0.107	0.334	< 0.001
Swing phase (%GC)	0.329	0.196	0.463	0.020	0.397	0.256	0.538	0.163	**0.772**	**0.657**	**0.887**	** < 0.001**
Double support phase (%GC)	0.685	0.554	0.817	0.011	0.597	0.456	0.739	0.188	0.220	0.107	0.334	< 0.001
Step time (s)	0.632	0.493	0.770	0.073	0.528	0.382	0.674	0.704	0.339	0.202	0.476	0.030
Stride time (s)	0.629	0.490	0.767	0.080	0.531	0.385	0.676	0.678	0.340	0.203	0.477	0.032
Cadence (steps/min)	0.379	0.240	0.518	0.099	0.479	0.334	0.625	0.778	0.647	0.508	0.785	0.049
Gait Speed (m/s)	0.287	0.158	0.416	0.004	0.356	0.218	0.493	0.051	**0.823**	**0.715**	**0.930**	** < 0.001**
**Dual-task walking:**												
Foot rotation angle (deg)	0.609	0.464	0.755	0.155	0.557	0.408	0.707	0.439	0.312	0.164	0.459	0.016
Step length (cm)	0.347	0.201	0.493	0.046	0.291	0.162	0.420	0.005	**0.813**	**0.695**	**0.930**	** < 0.001**
Stride length (cm)	0.347	0.201	0.493	0.046	0.291	0.162	0.420	0.005	**0.813**	**0.695**	**0.930**	** < 0.001**
Step width (cm)	0.603	0.454	0.753	0.179	0.692	0.560	0.824	0.010	0.238	0.113	0.363	0.001
Stance phase (%GC)	0.654	0.510	0.797	0.045	0.633	0.495	0.772	0.071	0.192	0.075	0.309	< 0.001
Loading response (%GC)	0.658	0.516	0.800	0.040	0.624	0.485	0.764	0.092	0.190	0.072	0.307	< 0.001
Single support phase (%GC)	0.316	0.179	0.452	0.016	0.376	0.237	0.515	0.094	**0.822**	**0.713**	**0.931**	** < 0.001**
Pre-swing (%GC)	0.656	0.514	0.799	0.042	0.629	0.490	0.768	0.081	0.217	0.094	0.340	< 0.001
Swing phase (%GC)	0.346	0.203	0.490	0.045	0.367	0.228	0.505	0.071	**0.808**	**0.691**	**0.925**	** < 0.001**
Double support phase (%GC)	0.659	0.516	0.801	0.039	0.625	0.486	0.764	0.091	0.213	0.090	0.335	< 0.001
Step time (s)	0.636	0.493	0.780	0.076	0.613	0.472	0.753	0.128	0.254	0.128	0.381	0.002
Stride time (s)	0.638	0.494	0.781	0.073	0.615	0.474	0.755	0.121	0.252	0.126	0.378	0.001
Cadence (steps/min)	0.371	0.226	0.516	0.094	0.395	0.254	0.536	0.155	**0.746**	**0.619**	**0.873**	**0.002**
Gait Speed (m/s)	0.324	0.183	0.465	0.022	0.319	0.187	0.451	0.014	**0.823**	**0.711**	**0.935**	** < 0.001**

%GC: Percent of gait cycle; Bold indicated significantly groups classification with acceptable accuracy level (≥ 0.7 of AUC)

## Discussion

No differences in demographic data were seen between the three groups, except for age, year of education, MoCA, and hypertension. The majority of participants in the MCI and dementia groups were female which was consistent with the findings of a previous systematic review [61]. Our findings revealed that the dementia group had the highest average age, which is not surprising given that the disease is more common in older people and its prevalence increases with age [1, 61]. Individuals with AD who participated in our study were in the mild to severe stages with a large range of MoCA scores from 8 − 20. Considering the educational factor, it was not surprising that the dementia group had the lowest level of education. As reported previously, individuals with a higher level of education were more able to maintain cognitive ability than those with a lower level [62].

Among the comorbidities tested in this study, we found that MCI and dementia groups were more afflicted with hypertension and diabetes mellitus than the cognitively intact group. Hypertension is widely known as a major risk factor of damage to several organs, including the brain. Blood pressure variability and 24-h blood pressure profiles have been linked to cognitive impairment and/or silent cerebral diseases, such as silent cerebral infarction or white matter lesions, both of which are risk factors for cognitive impairment and dementia [63]. A certain number of MCI and dementia groups were diagnosed with diabetes mellitus (*n* = 9 or 31% and *n* = 7 or 25%, respectively) and cerebrovascular disease (*n* = 6 or 21% and *n* = 3 or 11%). Type 2 diabetes mellitus raises the risk of vascular dementia and Alzheimer's disease. The underlying mechanisms include the increase in neuronal insulin resistance, impaired insulin signaling, pro-inflammatory state, mitochondrial dysfunction, and vascular damage. All of which increase the deposition of β-amyloid, tau proteins, and GSK3β, leading to an earlier onset of dementia [64].

We found a decrease in gait performance in individuals with cognitive decline when compared with those who were cognitively intact. Specifically decreases in step length, stride length, single support phase, cadence, and gait speed; and increases in stance phase, loading response, pre-swing, double support, step time, and stride time. However, there was no change in the foot rotation angle and step width. Similar to previous findings, these measures of decline in gait performance were noticeable during single-task walking but showed greater deterioration when tested under a dual-task walking condition [36, 40, 65–67]. From our findings, there were significant differences in several gait variables between the MCI and dementia groups and between dementia and cognitively intact groups when tested under single- and dual-task walking. However, there were no differences in gait variables between the MCI and cognitively intact groups, whether testing under single- or dual-task walking. This may be the result of several factors, including 1) differences in the sample criteria and classification, 2) variability of the data observed by a larger standard error, even though the mean differences were similar between the groups, 3) dual-task walking test procedure, in which counting backward with one might challenge cognitive function to a lesser extent than other counting tasks such as subtracting by seven [68]. Subtraction with one was chosen in this study because the technique can be used to assess the majority of individuals with dementia. Although the method seems to be very simple, there were five individuals with dementia unable to perform this task. However, our counting method may not cognitively challenge a large number of individuals in the MCI group, thus the results cannot be differentiated from the cognitively intact group. A recent systematic review with meta-analysis. reported that the spatiotemporal gait variables (e.g., gait speed, cadence, stride length, stride time, stride time variability, and stance time) could be used to classify normal and dementia groups when tested using a single task. In addition, the dual-task could differentiate between these two groups using gait speed, stride length, and stride time variability only [69]. Another point of consideration is that the mean values of the gait variables in our study were substantially different from previous reports from Western countries, which may be due to racial, anthropometric, and sociocultural differences.

For the efficacy of using gait variables to compare individuals with different cognitive statuses, all gait variables except foot rotation angle showed significant differences between groups when testing gait under the dual-task condition. When testing with the single-task condition, most gait variables showed discriminating ability between groups, except foot rotation angle, step width, step time, stride time, and cadence. Based on the effect size criterion using the partial Eta square (η^2^_*p*_), which are defined as small (> 0.01), medium (> 0.06), and large (> 0.14) effects. Step length, stride length, and gait speed had large effect sizes when assessed under both the single- and dual-task walking, while other variables (stance phase, loading response, single support phase, pre-swing, swing phase, and double support phase) had medium effect sizes when assessing under the single- and dual-task walking. In addition, medium effects were found in step width, step time, stride time, and cadence when tested under dual-task walking. This was consistent with several previous studies that reported a significant reduction in gait performance as evidenced by reduced step or stride length and reduced gait speed in individuals with cognitive decline compared to healthy controls [24, 39, 40, 66, 67, 70–72]. In addition, gait performance was more impaired in the MCI group with episodic of memory decline [41, 71, 73]. However, the differences among studies were due to the characteristics of the participants, equipment, and procedure. For instance, the study done by Zhou et al. used a body-worn system to determine the most sensitive gait parameter in identifying older adults with and without cognitive frailty. The largest effect size was observed with dual-task gait speed with Cohen’s effect size d of 0.97, *p* < 0.001 only [67]. In contrast, another study reported that gait measures during a dual-task gait were not indicative of cognitive impairment in individuals with Parkinson’s disease [74].

Comparing gait data between single- and dual-task in each group, we found that walking abilities in all groups declined with the cognitive challenge of counting backward by one. Differences were found in most gait variables in both MCI and dementia groups. During the test, we observed that the dementia group demonstrated balance impairments (e.g., unsteady on feet, body swaying). Many of them had an obvious sway during gait with a markedly slower walking speed. This was confirmed by an increase in the double support phase when testing under the dual-task when compared to the single-task, which was 3.25 times higher in the dementia group compared to the cognitively intact group. Likewise, the MCI group was similarly altered and found to be 2.53 times higher than the cognitively intact group. Challenging the brain function by subtracting a number with one while walking seems easy for cognitively intact individuals. However, we found that cognitively intact individuals were still able to preserve function without modifying the phase-related gait variables, although cadence, temporal, and gait speed were found to decrease during the dual-task in this group.

In classifying the groups using the AUC test on different single gait variables as well as the combined variables. The dementia group was differentiated from the cognitively intact group by step length, stride length, single support phase, swing phase, and gait speed under the single- and dual-task walking tests. Cadence can also be used to differentiate these two groups if tested under the dual-task walking condition. Additionally, using the multiple computed gait variables can also be used to differentiate between dementia and cognitively intact groups as well as between the MCI and dementia groups. However, we did not find any variables which could classify the MCI group from the cognitively intact group.

Two previous studies used a wearable tool to differentiate gait in individuals with and without cognitive impairment [67] and among subtypes of dementia [75]. Zhou et al. reported that the single and dual-task gait speed could be used to differentiate cognitive frailty individuals with moderate accuracy (AUC of 0.73 for single-task and 0.76 for dual-task) [67]. MCI or dementia due to AD, dementia with Lewy bodies (DLB), and PD were able to be differentiated using 14 gait characteristics representing pace, variability, rhythm, asymmetry, and postural control [75]. Whereas this current study identified that all single gait variables, except foot rotation angle, showed the potential to differentiate cognitive statuses under both single and dual-task conditions.

This current study has some limitations as details of variability and asymmetry of gait characteristics were not determined. Gait variables should be analyzed in more diverse perspectives, such as intrapersonal gait variability and asymmetry or the difference between single- and dual-task. Although this study highlights interesting findings a longitudinal study should be performed to obtain a clearer link between the gait parameters and cognition. Comparisons of the data between different types of dementia as well as studying the relationship and underlying mechanism or process between gait and cognition through the mediator should be explored in the future.

## Conclusions

Almost all single gait variables, except foot rotation angle and step width, showed the potential to differentiate between cognitive status under both single and dual-task conditions. The largest effects were found in step length, stride length, and in particular gait speed during both walking conditions. Gait speed is therefore recommended to be used to determine early clinical signs of people with cognitive problems, which should improve the accuracy of such diagnostic assessments and highlight the need for early management.

## Data Availability

The datasets generated and/or analyzed during the current study are not publicly available due to limitations of ethical approval involving the patient data and anonymity but are available from the corresponding author on reasonable request.

## Abbreviations

MCI: Mild cognitive impairment
AD: Alzheimer's disease
DALYs: Disability-adjusted life-years
DSM-5: Diagnostic and Statistical Manual of Mental Disorders, 5^th^ edition
MoCA: Montreal Cognitive Assessment
FDM: Force Distribution Measurement
AUC: Area under the curve
ROC: Receiver operating characteristic
